# Measuring Liquid-Level Utilizing Wedge Wave

**DOI:** 10.3390/s18010002

**Published:** 2017-12-21

**Authors:** Iwao Matsuya, Yudai Honma, Masayuki Mori, Ikuo Ihara

**Affiliations:** Department of Mechanical Engineering, Nagaoka University of Technology, Niigata 940-2188, Japan; s133073@stn.nagaokaut.ac.jp (Y.H.); s143086@stn.nagaokaut.ac.jp (M.M.); ihara@mech.nagaokaut.ac.jp (I.I.)

**Keywords:** liquid-level, wedge wave, ultrasound, FEM simulation

## Abstract

A new technique for measuring liquid-level utilizing wedge wave is presented and demonstrated through FEM simulation and a corresponding experiment. The velocities of wedge waves in the air and the water, and the sensitivities for the measurement, are compared with the simulation and the results obtained in the experiments. Combining the simulation and the measurement theory, it is verified that the foundation framework for the methods is available. The liquid-level sensing is carried out using the aluminum waveguide with a 30° wedge in the water. The liquid-level is proportional to the traveling time of the mode 1 wedge wave. The standard deviations and the uncertainties of the measurement are 0.65 mm and 0.21 mm using interface echo, and 0.39 mm and 0.12 mm utilized by end echo, which are smaller than the industry standard of 1.5 mm. The measurement resolutions are 7.68 μm using the interface echo, which is the smallest among all the guided acoustic wave-based liquid-level sensing.

## 1. Introduction

In order to detect fluid capacity, a liquid-level measurement has been carried out in the several fields such as fuel tanks, water cisterns, and so on [1,2,3]. For a fuel tank in the automobile and motorcycle industry in particular, it is important to accommodate a request from more complex tank geometry whose height is getting shallower to ensure safety for a potential ignition and to be robust against pollution. Therefore, a high measurement resolution, a high level of safety, reduced need for infrequent maintenance, and cost-effectiveness are strongly demanded for liquid-level sensing in automobiles [4,5,6]. There are many methods that can be used to measure the liquid level, some of which are the float type, the electro-static type [7,8], the optical type [9], optical fiber sensors [10,11,12,13], the guide pulse type [14], and the ultrasonic type [11,12,13]. The float type detects the position of the float on a liquid surface combined with a resistive sensor, which has been used since early times because of its simple structure and reasonable cost. The electro-static type utilizes the electric capacitance change between a coaxial cable and a reservoir containing a liquid due to an increase in the liquid-level. Even a high viscosity liquid can be measured by this type as long as the conductive property of the liquid is known. The optical sensor, including a commercially available laser distance sensor, consists of light emitters and photo detectors, which detect the light intensity and the position of a light spot with a high level of accuracy according to the position of the liquid surface. One of the fiber sensors has grooves, and the difference in the refractive index between the air and the liquid, which is similar to the fiber core, is taken advantage of [10]. If the liquid-level increases, the signal dissipation is reduced due to the contact of the liquid with the core. Although it has a resolution of only 20 cm, because this technique depends on the entry of water into the fiber core through the grooves, it can be applied in large-scale structural health monitoring (SHM). The other fiber sensors utilize Fiber Bragg Grating (FBG) [11], the response of Raman fiber scattering between different heated mediums [12], and the self-imaging properties of the multimode interference (MMI) [13]. Those sensors offer numerous merits, such as resistance to corrosion, robustness against noises, high accuracy, and so on. The guide pulse type uses the reflection of electric pulses at the liquid surface, which accomplishes both high precision and maintenance free requiments. However, the float type suffers from low accuracy, and the electro-static type cannot be operated if the temperature changes widely, which leads to large permittivity change; the optical type requires frequent maintenance, the performance of the fiber sensors is excessive, and it is not cost-effective to apply them in automobile fuel tanks, and the guide pulse type runs the risk of having an electric spark. 

The ultrasonic type is the method that detects the liquid-level by elapsed time of ultrasound-utilizing, air-coupled ultrasound [15,16], the immersed-type ultrasonic sensor [17,18], and guided ultrasound [19,20,21,22]. Those are widely employed in many applications because of their simplicity of use and safety. In the case of using air-coupled and immersed ultrasound, maintenance on a regular basis is required. On the contrary, the guided ultrasound method demands only minimum maintenance, and it can be operated robustly for a long time because the resource of ultrasound does not need to be exposed in the liquid tank. There are some kinds of guided ultrasound such as surface wave [19], torsional wave [21,22], transverse wave [5], and so on. We focused on the wedge wave in particular. A wedge wave is a special kind of a surface acoustic wave that propagates along the ridge of a wedge one-dimensionally [23,24,25]. Since the energy of that is concentrated within the region that corresponds to almost its entire wave length at the tip of a wedge, the wedge wave can travel long distances with a low attenuation [26]. It vibrates asymmetrically and has low velocity dispersion [27,28,29]. In addition, the velocity of a wedge wave is relatively slow compared to the other guided waves, so it is considered that the measurement accuracy, which is closely related to the time resolution of measurement equipment, can be improved upon. Besides, when the waveguide where the wedge wave propagates is immersed in water, its velocity is reduced [30,31,32]. However, behavior of its propagation is not well-understood, because the wedge wave does not have analytical solutions.

In this paper, a new method for liquid-level measurement utilizing the wedge wave is proposed and experimentally demonstrated. First, the measurement theory of the liquid-level-sensing, utilizing wedge wave is shown. Second, a numerical simulation is conducted on the propagation behavior of the wedge wave to investigate the velocities of that in the air and the water, as well as on the sensitivities for the measurement methods. Third, the liquid-level is experimentally detected using the aluminum waveguide rod with 30° wedge. Finally, the resolution and the accuracy of the method is investigated, and the feasibility of this method for actual use is verified.

## 2. Measurement Theory 

**Figure 1:**
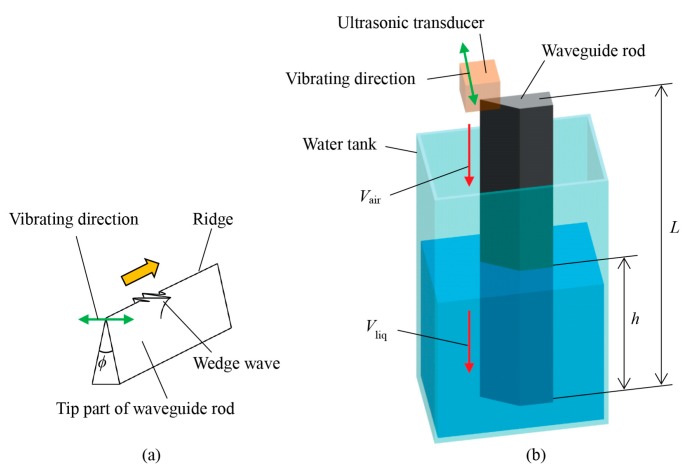
Schematic depiction of liquid-level measurement utilizing wedge wave on waveguide rod. (**a**) Enlarged view of the tip part of the waveguide rod where wedge wave is propagating along the ridge of the wedge. (**b**) Experimental setup. Except for the waveguide, all the components are displayed transparently for visibility. Wedge waves are generated by the ultrasonic transducer. After the launch, wedge waves propagate at *V_air_* in the air and *V_liq_* in the water.

Figure 1 shows the schematic depiction of the liquid-level sensing. The waveguide rod with a sharp wedge, whose length is *L*, is located vertically in the water tank with liquid-level *h*, as shown in Figure 1b. The ultrasonic transducer for shear wave is attached to the front face of the waveguide, and a wedge wave is generated. The relationship between the vibrating direction and the propagated wedge wave is shown in Figure 1a. The wedge wave is reflected at the surface of the water and at the bottom of the waveguide, which are measured at the top of the waveguide by the identical ultrasonic transducer. The velocity of the wedge wave in the air *V_air_* is expressed using the velocity of Rayleigh wave *V_R_* and wedge angle ϕ as follows [33],

(1) $$V_{air}=V_{R}\sin\left(m\phi \right).$$

where *m* is the order of wedge wave mode. For example, since the velocity of Rayleigh wave for the waveguide rod is experimentally obtained 2940 m/s, the velocities of mode 1 and 2 wedge wave are estimated to be 1470 m/s for mode 1 and 2546 m/s for mode 2, respectively. Note that since Equation (1) is an empirical formula, it is expected that *V_air_* derived from Equation (1) is slightly different from the experimental value [34]. There are two methods to measure the liquid-level; one is the interface echo method, and the other is the end echo method. The interface echo method simply utilizes the reflected wedge wave from the liquid surface. If the liquid-level is zero, using the velocity of wedge wave in the air *V_air_*, the elapsed time *t*_0_ is shown below:

(2) $$t_{0}=2L/V_{air}$$

The relationship between the liquid-level and the elapsed time difference of a wedge wave reflected at the liquid surface is expressed as follows,

(3) $$h=-\frac{V_{air}}{2}\Delta t,$$

where *V_air_* is the velocity of wedge wave in the air, and Δ*t* is the traveling time, which is the total elapsed time of wedge wave minus *t*_0_. On the other hand, the velocity of the wedge wave in the water is slower than that in the air because of the fluid loading. The end echo method utilizes the wedge wave reflected from the bottom of the waveguide rod. The higher the liquid-level is, the longer the elapsed time of the wedge wave propagated via the bottom is. Consequently, if the elapsed time of the wedge wave is measured, we can detect the liquid-level. If the liquid-level is *h*, the total elapsed time *T* is expressed as follows,

(4) $$T=\frac{2\left(L-h\right)}{V_{air}}+\frac{2h}{V_{liq}},$$

where *V_liq_* is the velocity of a wedge wave in the water. Using Equations (2) and (4) and the relation of the traveling time Δ*t* = *T* − *t*_0_, the liquid-level for the end echo method is derived as follows,

(5) $$h=\frac{V_{air}\;V_{liq}}{2\left(V_{air}-V_{liq}\right)}\Delta t.$$

Note that the sensitivity of both methods is equal to the proportionality coefficient between liquid-level and traveling time. For the interface echo method, the slower the velocity of wedge wave in the air is, the better the sensitivity becomes as shown in Equation (3). For the end echo method, the bigger the gap between the velocity of a wedge wave in the air and the water, the greater the sensitivity, as shown in Equation (5).

## 3. Experiments

### 3.1. Numerical Simulation

**Figure 2:**
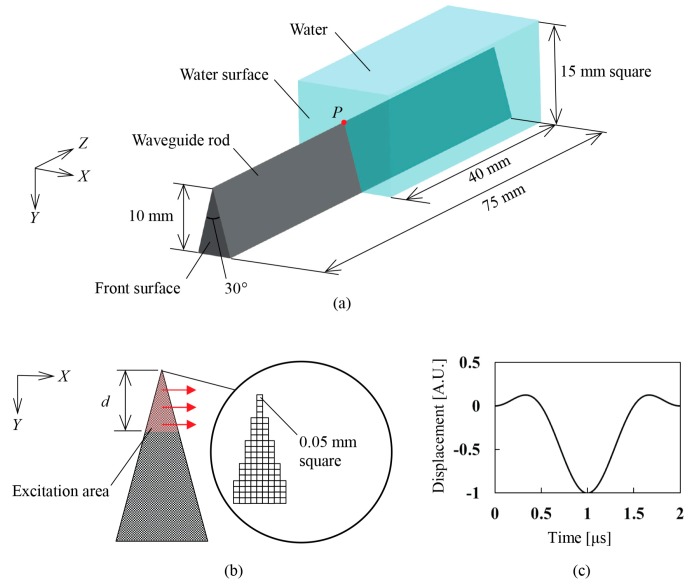
Schematic diagrams of simulation model. (**a**) Waveguide rod with sharp wedge attached to the water. (**b**) Excitation area at the front surface of the waveguide rod and finite element meshes near the tip of the waveguide rod. (**c**) Input waveform for excitation of wedge wave at the front surface.

The suggested end echo method is based on the velocity change of wedge wave in the air and liquid as mentioned above. To verify the propagating behavior of wedge wave in those mediums, a three-dimensional finite element method (FEM) is performed with the software ComWave (ITOCHU Techno-Solutions Corporation, Tokyo, Japan). Figure 2a shows a geometrical shape and a positional relation of the waveguide rod with a sharp wedge and the water. The waveguide model has a sharp wedge whose angle is 30°; this sharp wedge is 75 mm in length and 10 mm in height. The waveguide is partially covered with the water area, which is 40 mm in length from the middle of the waveguide to the end of it. The velocities of longitudinal and shear wave for aluminum waveguide are set to be 6330 m/s and 3150 m/s, respectively. The sound velocity in the water (longitudinal wave) is set to be 1480 m/s. The densities of aluminum and water are 2700 kg/m^3^ and 998 kg/m^3^. The excitation area is arranged to be near the tip on the front surface of the waveguide where the excitation displacement is applied in *X*-direction, as shown in Figure 2b. To ensure successful and accurate wave propagation analysis, the voxel element size was decided to be 50 μm on a side, which is less than 1/40 of the shortest wavelength of interest. The length *d* of the excited area is arranged to be 2.8 mm at first and is changed to examine the influence of the *d* on the wedge wave propagation. In Figure 2c, the input waveform for an excitation of a wedge wave is decided to be a typical Mexican hat function at the frequency of 0.5 MHz, which implies that the electrical square pulse is applied to the ultrasonic transducer for a shear wave attached to the waveguide in an actual experiment. Following the excitation, a wedge wave is generated and propagated along the ridge of the waveguide rod. The behavior of the wedge wave propagation with and without water is investigated, and velocities in those mediums are compared with actual measured values. In addition, the sensitivities derived from those simulated velocities and Equations (3) and (5) are calculated, and the expected performance for the measurement methods are discussed. Incidentally, considering inclinations in the water surface to the waveguide is unavoidable for practical use. As shown in Figure 2a, the intersection point between the water surface and the ridge of the waveguide is defined as the fixed-point *P*, which is 35 mm away from the front surface of the waveguide. When the water surface on *P* is inclined ±45° around the *X*-axis and 45° around the *Y*-axis, in accordance with the right-handed screw rule, the velocity of wedge wave and the propagation time in the air and the water are investigated through the FEM simulation to clarify the effect on the accuracy of the measurement. 

### 3.2. Measurement of Liquid-Level

**Figure 3:**
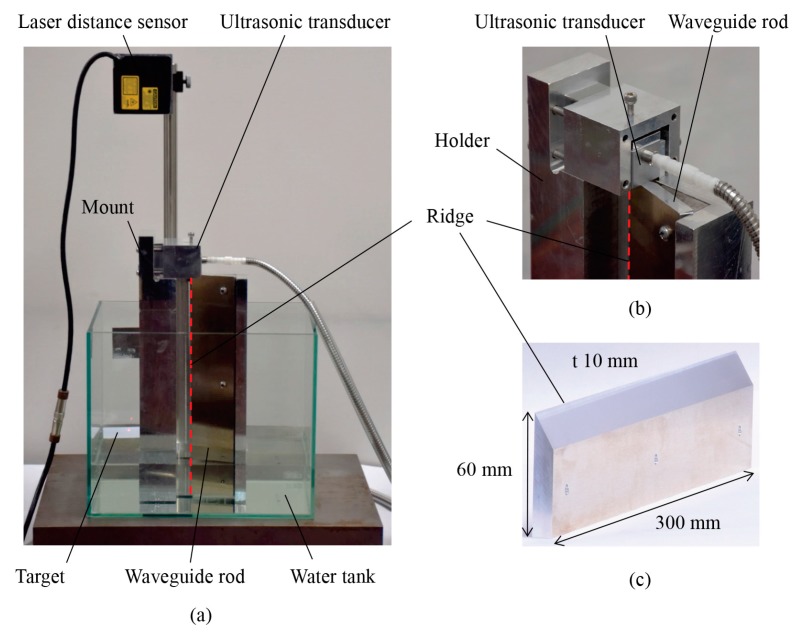
(**a**) Picture of experimental setup for measuring liquid-level. (**b**) Contact part of ultrasonic transducer and waveguide rod. (**c**) Waveguide rod. Red dashed line indicates ridge of waveguide rod.

Liquid-level is measured by both the interface echo method and the end echo method. Figure 3 shows pictures of the experimental setup. The waveguide rod is placed vertically in the water tank, and the ultrasonic transducer (0.5Z20 × 20SN, shear wave type, 26 mm square, 0.5 MHz for central frequency, Japan probe Company Limited, Yokohama, Japan) is immobilized at the top of the waveguide using a mount. The target made of plastic is floated on the surface of the water, and its height is measured by a laser distance sensor (CD5-W200, 0.02 μm resolution, OPTEX FA Company Limited, Kyoto, Japan), which is used as an absolute reference, as shown in Figure 3a. The waveguide of 300 mm length, 60 mm width, and 10 mm thickness, as shown in Figure 3c, is made of aluminum (A5052) and has a wedge angle of 30°, the sharpness of which is approximately 0.04 mm. Although the cross-section shape of the waveguide (trapezoidal shape) is macroscopically different from the simulation model (triangular shape) as shown in Figure 2, it is considered that the behavior of the wave propagation is not affected by the cross-section shape of the waveguide, because wedge wave propagates from a small area at the tip of the waveguide. As shown in Figure 1b and Figure 3b, the center of the ultrasonic transducer is attached to the end of the ridge and its vibrating direction is vertically arranged against the angle bisector of the wedge. Note that even if the vibrating direction is inclined to the wedge within around 15 degrees, the obtained waveforms showed little difference. Liquid-level is changed from 0 to 180 mm in increments of 10 mm. The ultrasonic transducer is driven by an electrical pulse signal from a square-wave pulsar with a repetition rate of 500 Hz. When the wedge waves are received, the ultrasonic vibration is converted into voltage signals inside the ultrasonic transducer. The voltage signals are captured using a 12-bit serial acquisition board at a sampling rate of 100 MHz. In order to reduce the background noise of the signals, the bandpass filter from 200 to 400 kHz is utilized, and the averaging procedure is conducted from 50 acquired signals. The liquid-level detections are carried out ten times to obtain standard deviations of the measurement. To avoid wind disturbance, the water tank is covered with a plastic wrap, and the air in and out of the tank is minimized. As shown in Figure 3, the experimental setup is placed on the stabilizer panel made of steel, and the vibrational disturbance is carefully prevented. As a result, the laser distance meter indicates a fluctuation of 0.001 or 0.002 mm during the experiment. When the velocities of the propagated wedge wave are derived from the waveform, the cross-correlation method is utilized to obtain the elapsed time by identifying each wave of interest. The resolution of the liquid-level measurement is obtained by the change of the traveling time of the wedge wave according to the liquid-level and the sampling rate of the measurement device. From the viewpoint of measurement accuracy and sensitivity, the feasibility of the methods for actual use is discussed.

### 3.3. Effect of Wet Surface

This method utilizes the velocity reduction when transmitting from the air into the liquid. Therefore, when the surface of the waveguide is wet, we have to investigate what the effect on measurement accuracy is expected. First, the waveguide rod is soaked in water and is picked up to the air, and the velocity of the wedge wave is measured. This is repeated 5 times. Second, the waveguide rod is entirely covered with small drops of water using a water spray, 40 cm away from it, and the velocity measurement is conducted as well. For the purpose of practical application, the effects of a wet surface are explored.

## 4. Results and Discussion

### 4.1. Numerical Simulation

**Figure 4:**
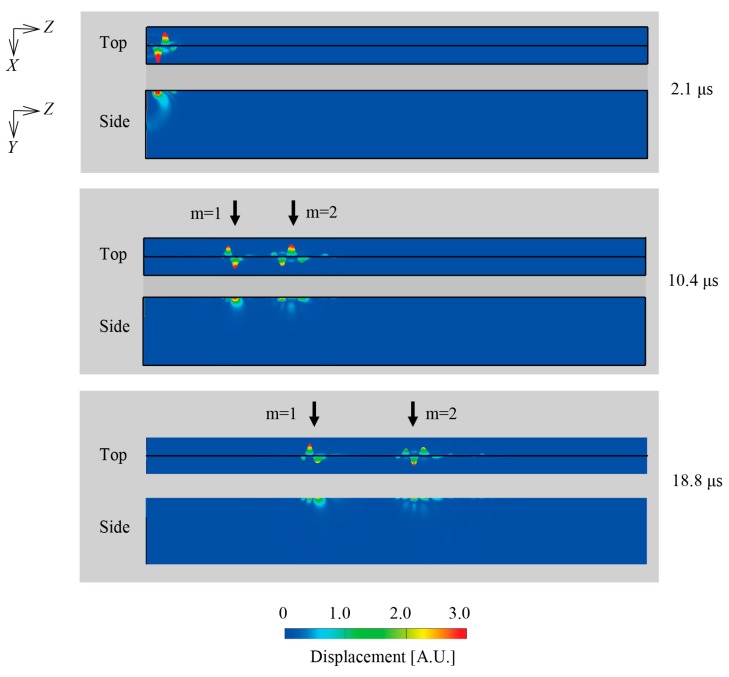
Simulation results of wedge wave propagating in the air. Top and side view of the waveguide is shown from 2.1 to 18.8 μs. The colored waveform represents degrees of displacement. The faster wedge wave is mode 2 and the slower one is mode 1.

Figure 4 shows the simulation results of the propagated wedge wave in the air. The color bar represents the displacement of the excited waves. The acoustic vibration is confined near the tip of the waveguide and is propagated along the ridge of the wedge in *Z*-direction, predictably. At the time of 2.1 μs, the mode 1 and 2 wedge waves are almost mixed near the front surface of the waveguide. As time advances, it is clearly observed that two wave packets arise and propagate at different velocities. The faster wave packet is considered to be the mode 2 wedge wave because two peaks of the displacement can be seen across the ridge from each other on the cross-section (*XY*-plane) of the waveguide. On the other hand, a slower wave packet is expected to be a mode 1 wedge wave because of the only peak on the cross-section. The observed results have the characteristics like the asymmetrical bending vibration of cantilever beam for mode 1 and 2 [35]. After enough time passes, the vibrated region of the waveguide for both the mode 1 and 2 is estimated to be roughly 3 mm vertical to the ridge, which is comparable with the wave length of the wedge waves. Note that almost similar results are obtained as long as the excitation length *d* is set to be around 3 mm. The velocities of mode 1 and 2 wedge waves through the simulation are 1424 m/s for mode 1 and 2330 m/s for mode 2, respectively. The velocities obtained by experiment are 1528 m/s for the slower wave packet and 2441 m/s for the faster one. Although the simulated values are a little smaller than the experimental values within 7%, the wedge waves in the air are successfully generated and propagated, and the behavior of its propagation is demonstrated in the simulation. 

**Figure 5:**
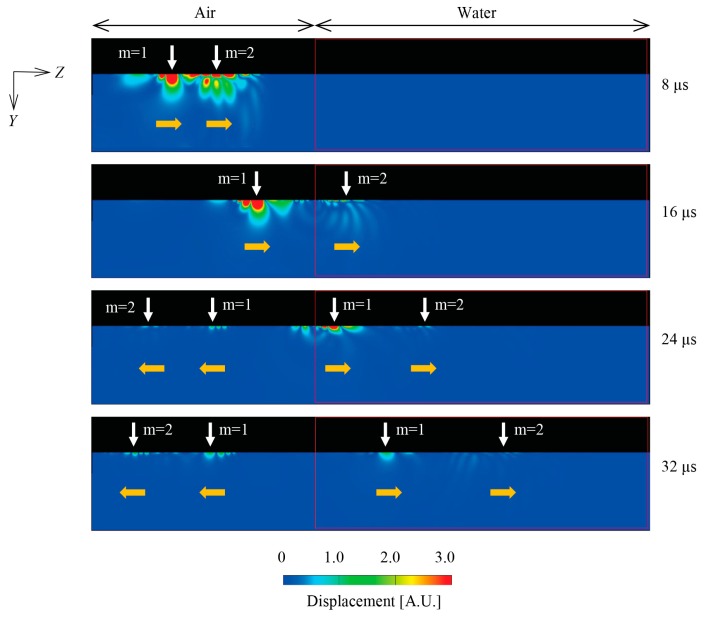
Simulation results of wedge wave traveling from the air to the water. Side views of the waveguide are shown from 8 to 32 μs. The reflected mode 1 and mode 2 wedge waves propagate to the left at 24 μs and 32 μs.

Figure 5 shows the simulation results of a wedge wave propagated from the air to the water. Side views of the waveguide are shown from 8 to 32 μs. At the time of 8 μs, the wave packets in the air begin to split into mode 1, which has only displacement peak, and the mode 2, which has two peaks on the cross-section, as depicted above. When the mode 2 wedge wave propagates into the water at 16 μs, the velocity of that is diminished to 1441 m/s. Moreover, the mode 2 wedge wave is reflected back into the air and is partially converted to mode 1 at the surface of the water. At 24 μs, the reflected mode 1 that is converted from the original mode 2 and the reflected mode 2 wedge waves propagate to the left (−*Z*-direction) in the air. Besides, the velocity of mode 1 is reduced to 962.6 m/s in the water. After that, the reflected mode 1 and mode 2 wedge waves travel to the left in the air at 32 μs, which originate from mode 1 as well. The velocities in the water obtained by experiment are 1076 m/s for mode 1 and 1263 m/s for mode 2. Those results are summarized in Table 1. 

The simulated mode 1 velocity is 10.5% smaller than the experimental value, whereas the simulated mode 2 value is 14.8% bigger than the experimental value. However, the velocity reductions from the air to the water are 29.5% (experiment) and 32.4% (simulation) for mode 1 and 48.3% (experiment) and 38.2% (simulation) for mode 2, which denote a similar tendency. Although the amplitudes of not only the mode 2 wedge waves but also mode 1 in the water are considerably decreased, which is different from the experiment, the velocities of wedge waves are basically obtained and the perspective of propagation behavior for both the interface and the end echo methods are clarified. The framework of the measurement method can be reproduced by combining the measurement theory and the simulation. Using Equations (3) and (5), and velocities obtained by the simulation, the gradient between the traveling time and the liquid-level can be predicted, which is regarded as the sensitivity of the measurement. The simulated values of the sensitivity for the interface echo method are −0.712 mm/μs and −1.165 mm/μs for mode 1 and 2, and those for the end echo method are 1.485 mm/μs and 1.888 mm/μs for mode 1 and 2. In comparison, the sensitivities for the interface echo method using the velocities obtained in the experiment are −0.764 mm/μs and −1.220 mm/μs for mode 1 and 2, and those for the end echo method are 1.818 mm/μs and 1.309 mm/μs for mode 1 and 2. Those values are summarized in Table 2. 

The simulated sensitivities for the interface echo method agree well with the sensitivities derived by the experimental velocities, whereas the simulated sensitivities for the end echo method are appreciably different from the sensitivities derived by the experimental velocities. It is considered that since the parameters regarding viscosity, hydrophily, hydrophobicity, contact, and so on are not taken into account in the simulation, the velocities of the wedge wave in the water cause the differences between the simulation and the experiment. 

When the water surface on the point *P* in Figure 2 is inclined at an angle of 45° around the *X* and *Y* axes, the influence on the measurement accuracy is also investigated at the point of *Z* = 15 mm, which is 20 mm away from the water surface in the air, and *Z* = 55 mm, which is 20 mm away from the water surface. The reflected and the transmitted mode 1 wedge waves were observed at almost the same timing. In addition, the rates of velocity change for those waves were within 2%. Therefore, even if the water surface is inclined around the fixed-point on the ridge of the waveguide, the measurement accuracies for both methods are only slightly affected by them.

### 4.2. Measurement of Liquid-Level

**Figure 6:**
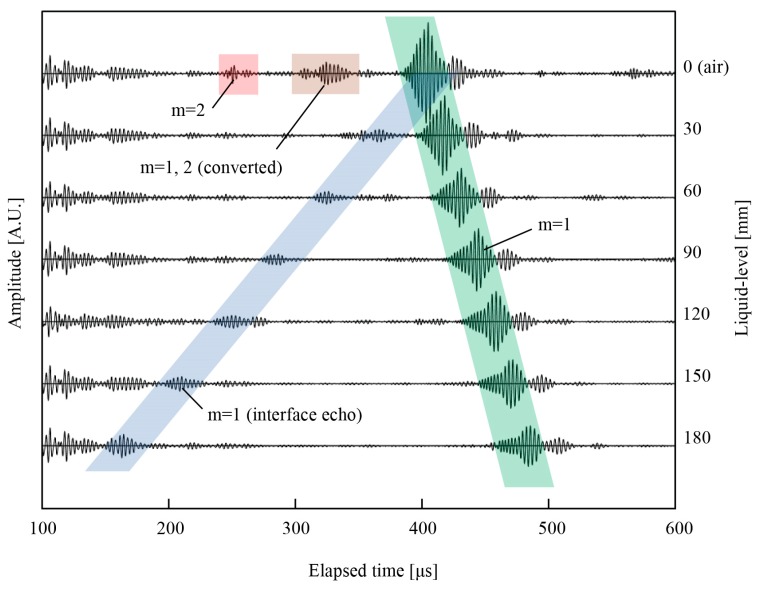
Waveforms of wedge waves for liquid-level from 0 to 180 mm. The red shaded part shows the wave packet of the mode 2 wedge wave in the air. The brown shaded part shows the wave packet of the mode 1 and 2 wedge waves, which are originally mode 2 and 1, respectively, at the end of the waveguide. The green shaded part shows the mode 1 wedge wave reflected from the end of the waveguide. The blue-colored shaded part indicates the mode 1 wedge wave reflected from the surface of the water.

Figure 6 shows waveforms of wedge wave according to the liquid-level of 30 mm step. The red shaded part shows the mode 2 wedge wave, which is rapidly diminished in the water, as shown in the waveforms of 0 and 30 mm liquid-levels. The brown shaded part shows the mode 1 wedge wave converted from mode 2 and the mode 2 wedge wave converted from mode 1, which are reflected at the end surface of the waveguide. Those are vanished in an instant due to the existence of the water, as well as to the case of the mode 2 wedge wave without mode conversion. The green-colored shaded part shows the wedge wave of mode 1 reflected from the end face of the waveguide, which is utilized for the end echo method. Although the signal-to-noise ratios for the mode 1 end echo are gradually reduced from 34.8 to 28.7 dB as the liquid-level increases, clear echoes can be obtained. The blue shaded part shows the reflected mode 1 wedge wave from the surface of the water, which is used for the interface echo method. The signal-to-noise ratio for the reflected mode 1 was 20.5 dB on average, which is an almost constant value even if the liquid-level increases and is still high enough for distinguishing from other waves. The reflected mode 1 wedge wave is obviously suitable for the measurement compared to the mode 2 because of its slowness and clear signal. At the beginning of each waveform, although there are noises which are waves propagating on the front surface of the waveguide, those are acceptable for the measurement because there is less signal mixing. The total elapsed time of the wedge wave reflected from both the end of waveguide and the surface of the water is gradually changed according to the liquid-level. Using Equations (3) and (5), and the traveling time obtained by the cross-correlation method, the liquid-level can be derived. 

**Figure 7:**
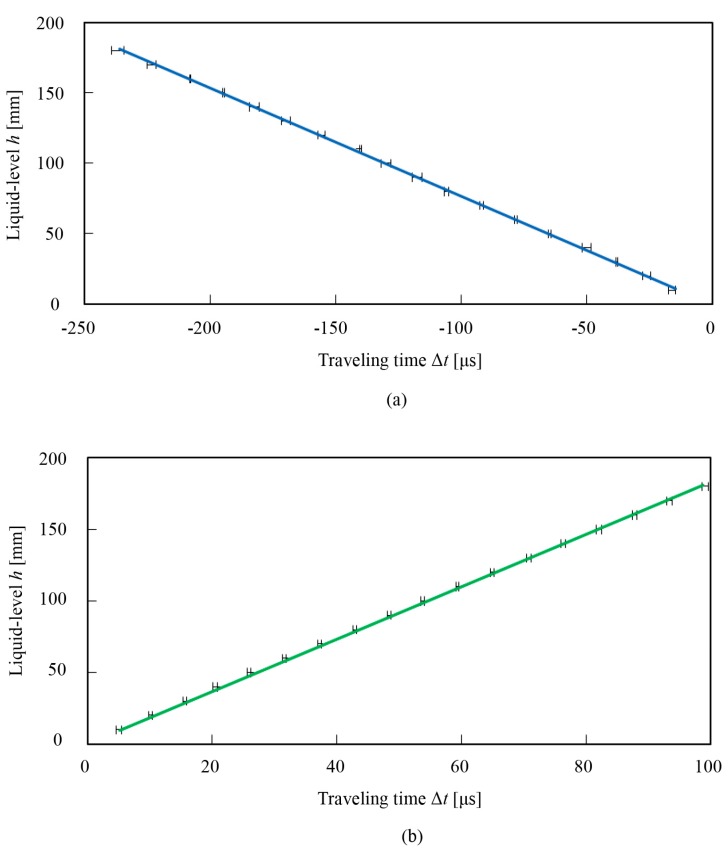
Relationship between liquid-level and traveling time for (**a**) the interface echo method and (**b**) the end echo method. The error bars are drawn for ten-time different measurements.

Figure 7 shows the traveling time with error bar according to the liquid-level for the interface echo method and the end echo method. The liquid-level is proportional to the traveling time of the propagated wedge wave. The relationship between the liquid-level and the traveling time of a wedge wave indicates high linearity. As shown in Equations (3) and (5), the gradient of the line corresponds to the sensitivity of the measurement. For the interface echo method, the gradient of the line is −0.768 mm/μs, and the averaged standard deviation of data plots is 0.65 mm as shown in Figure 7a. For the end echo method, the gradient is 1.83 mm/μs, and the averaged standard deviation is 0.39 mm as shown in Figure 7b. The combined standard uncertainties are 0.21 mm for the interface echo method and 0.12 mm for the end echo method [36,37]. In obtaining those values, the uncertainties for wedge wave velocities are ignored, which could affect the sensitivity of the measurement, because a 12-bit time-domain measurement is far more precise than the output of this measurement system. The experimental values of the sensitivity agree well with the sensitivities derived from the experimental velocities as shown in Table 2. Since the sampling rate of the measurement device is 100 MHz in this experiment, the measurement resolutions we obtained were 7.68 μm for the interface echo method and 18.3 μm for the end echo method. Both the standard deviations and the uncertainties for two methods, which are regarded as the measurement accuracy, are smaller than the industry standard of 1.5 mm [5,22]. The measurement resolution of the interface echo method is the smallest among all the acoustic guided wave based liquid-level sensing, and that of the end echo method is more than 10 times better than all the other end echo methods, to the best of our knowledge. It should also be added that although the mode 2 wedge wave is not used for the liquid-level measurement, the measurement resolutions for mode 2 derived from the experimental values of velocity are 12.2 μm for the interface echo method and 13.1 μm for the end echo method, which would be the best value among all the end echo methods. It is clear that the slowness of the wedge wave contributes to the high resolution for the interface echo method described in Equation (3), and the large gap in velocity between the air and the water also leads to high resolution for the interface echo method shown in Equation (5). The measurement range for the interface echo method is limited to 180 mm in this experiment because the signal mixing with the echo on the front surface of the waveguide at the beginning of the waveform starts at 180 mm in liquid-level, as shown in Figure 6. However, if the longer waveguide is prepared for the measurement, the signal mixing is prevented and the measurement range can be easily extended for both methods. Those results indicate that the measurement method is evaluated to be appropriate for actual use. 

### 4.3. Effect of Wet Surface

**Figure 8:**
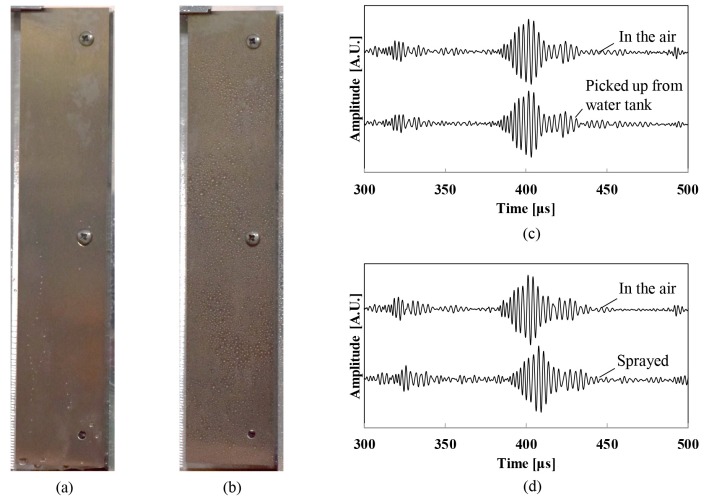
Effect of wet surface. (**a**) Picture of wet waveguide just after being picked up from the water and (**b**) after being sprayed. (**c**) Waveform of wedge wave in the air and just after being picked up from the water. (**d**) Waveform of wedge wave in the air and after water spray.

The waveguide is soaked in the water and is sprayed as shown in Figure 8a,b. The drop of water whose size is relatively big on the surface of the waveguide easily flowed down, because the waveguide originally has a water repellent surface, fortunately. Only small-sized drops of water can exist on the surface of the waveguide. Figure 8c shows the first echoes of the mode 1 wedge wave in the air just after being picked up from the water. Both waveforms agree well with each other. The velocity of the wedge wave in the air is 1526.0 m/s and the velocity just after being picked up from the water is 1526.1 m/s (averaged 5-times). Accordingly, if the size of the drop of water on the waveguide is relatively big, which implies the waveguide was submerged entirely and is picked up from the water, the accuracy of the liquid-level measurement is not affected at all because the velocities before and after soaking are almost identical. Besides, we investigated the case of small water droplets. The waveguide is filled with small-sized droplets over all, as show in Figure 8b. Figure 8d shows the first echoes of the mode 1 wedge wave in the air and just after spraying water. The arrival of the sprayed first echo is slightly delayed. The averaged value of the five tests of the wedge wave velocity just after being sprayed is 1514.2 m/s, the standard deviation of the velocity values is 1.54 m/s, and the reduction of the velocity compared to that in the air was 0.78%. As a result, if the size of the drop of water on the waveguide is relatively small, which implies the waveguide is exposed in the mist of the liquid, the effect on the accuracy of the measurement is insignificant because the velocity reduction is very small compared to that in the water. Furthermore, because instruments for removing or atomizing the drop of water can be easily invented and installed on the measurement equipment, it is considered that the wet surface of the waveguide will not significantly affect the accuracy of the measurement. 

## 5. Conclusions

The liquid-level measurement method utilizing wedge wave is formulated and demonstrated through the FEM simulation and the experiment for both the interface echo method and the end echo method. The simulated velocity values for mode 1 and 2 in the air and the water show a similar tendency with the experimental values, although there is some discrepancy between the experiment and the simulation. The large velocity reduction from the air to the water is confirmed in both the simulation and the experiment, which becomes the basis of the end echo method. The sensitivities for mode 1 and 2, and for the interface echo method, agree well with the sensitivities obtained by the experimental velocities of the wedge waves, whereas the sensitivities for mode 1 and 2, and for the end echo method, show considerable differences. However, it was demonstrated that if we utilize the simulation combining the measurement theory, the fundamental framework for the measurement method is available. Through the experiment, the mode 1 wedge wave is suitable for the liquid-level measurement compared to the mode 2 because of its slowness and clear signal in the water. The liquid-level is proportional to the traveling time of the mode 1 wedge wave for two methods. The interface echo method has a standard deviation of 0.65 mm and an uncertainty of 0.21 mm, and the end echo method has those of 0.39 mm and 0.12 mm, all of which are smaller than the industry standard of 1.5 mm. The measurement resolutions are 7.68 μm for the interface echo method and 18.3 μm for the end echo method, which are the smallest among all guided acoustic wave-based liquid-level sensing. This is not only due to the slowness of the wedge wave but also to the large velocity gap between the air and the water. The effects of the electric noise, the variation of the water surface, and the existence of water droplets are carefully investigated, and it is demonstrated those factors does not have much effect on the measurement accuracy. It is considered that the proposed method is characterized by low-cost, high safety, sufficient accuracy, high resolution, and infrequent maintenance for practical use.

## Figures and Tables

**Table 1:** Velocities of wedge wave in the air and the water.

	*V_air_* (m/s)			*V_liq_* (m/s)	
	Simulation	Experiment	Equation (1)	Simulation	Experiment
Mode 1	1424	1528	1470	962.6	1076
Mode 2	2330	2441	2546	1441	1263

**Table 2:** Sensitivities of liquid-level sensing.

	Sensitivity of Interface Echo Method (mm/μs)		Sensitivity of End Echo Method (mm/μs)	
	Simulation	Experiment	Simulation	Experiment
Mode 1	−0.712	−0.764	1.485	1.818
Mode 2	−1.165	−1.220	1.888	1.309
